# The Safety Evaluation of Salvianolic Acid B and Ginsenoside Rg1 Combination on Mice

**DOI:** 10.3390/ijms161226176

**Published:** 2015-12-09

**Authors:** Qun Zhao, Min Yang, Yanping Deng, Haitao Yu, Linlin Wang, Fukang Teng, Kenka Cho, Hongmei Ma, Peng Wu, Xue Li, Wanying Wu, Xuan Liu, Feng Xu, Baohong Jiang, De-An Guo

**Affiliations:** 1 Shanghai Institute of Materia Medica, Chinese Academy of Sciences, Haike Road #501, Shanghai 201203, China; zhaoqun886@163.com (Q.Z.); m_yang@simm.ac.cn (M.Y.); shimbiro@sina.com (Y.D.); yuhaitaojerry@163.com (H.Y.); hhxxwll@163.com (L.W.); fineskypig1983@163.com (F.T.); wp7665@sina.com (P.W.); shirley_lixue22@163.com (X.L.); wanyingwu@simm.ac.cn (W.W.); xuanliu@simm.ac.cn (X.L.); daguo@simm.ac.cn (D.-A.G.); 2 Shenyang Pharmaceutical University, Wenhua Road #103, Shenyang 110016, China; 3 Takarazuka University of Medical and Health Care, Hanayashiki-Midorigaoka, Takarazuka-City 6660162, Japan; duoli02@icloud.com; 4 East China University of Science and Technology Shanghai, Meilong Road 130, Shanghai 200237, China; hmma@ecust.edu.cn

**Keywords:** safety, salvianolic acid B, ginsenoside Rg1, acute toxicity, repeated toxicity

## Abstract

Our previous study indicated that the combination of salvianolic acid B (SalB) and ginsenoside Rg1 (Rg1), the main components of *Salvia miltiorrhizae* and *Panax notoginseng*, improves myocardium structure and ventricular function in rats with ischemia/reperfusion injury. The present study aimed to determine the safety of the combined SalB and Rg1 (SalB-Rg1) in mice. The safety of SalB-Rg1 was evaluated through acute toxicity and repeated-dose toxicity. In the acute toxicity study, the up and down procedure was carried out firstly, and then, the Bliss method was applied. In the toxicity study for seven-day repeated treatment of SalB-Rg1, forty Kunming mice were randomly divided into four groups. The intravenous median lethal dose (LD_50_) of the SalB-Rg1 combination was 1747 mg/kg using the Bliss method. For both the acute toxicity study and the seven-day repeated toxicity study, SalB-Rg1 did not induce significant abnormality on brain, heart, kidney, liver and lung structure at any dose based on H&E stain. There were no significant changes related to the SalB-Rg1 toxicity detected on biochemical parameters for two kinds of toxicity studies. The LD_50_ in mice was 1747 mg/kg, which was more than one hundred times higher than the effective dose. Both studies of acute toxicity and seven-day repeated dose toxicity indicated the safety of the SalB-Rg1 combination.

## 1. Introduction

The herb pair derived from the roots of *Salviae miltiorrhizae* (Danshen in Chinese) and *Panax notoginseng* (Sanqi in Chinese) has been widely used for improving coronary or cerebral circulation in China, as well as in Western countries [1,2]. Our previous study verified the significant cardio-protection for the combination of salvianolic acid B (SalB), the main active ingredient of *Salviae miltiorrhizae*, with ginsenoside Rg1 (Rg1), the main active ingredient of *Panax notoginseng*. Intravenous administration of the SalB-Rg1 combination at a ratio of 2:5 improves myocardium structure and ventricular function in rats with ischemia/reperfusion injury [3]. Either efficacy or safety is the precondition for new medicine development. Preliminary evaluation of toxicity becomes very necessary after elucidation of SalB-Rg1 on cardio-protection [4].

Fufang Danshen formulae, the commercially-available preparations including *Salviae miltiorrhizae* and *Panax notoginseng*, have been ranked as the first-line drugs among all Traditional Chinese Medicines in China [5]. More than 100 compounds have been isolated and identified in *Salviae miltiorrhizae* and *Panax notoginseng* to date, but only a fraction of these compounds were confirmed to be responsible for their biological effects [6,7]. Recently, an on-line coupled HPLC-DAD-ELSD method was successfully applied to the simultaneous quantification of multi-components [8]. It is unquestionable that the most abundant components among Fufang Danshen formulae are SalB, Rg1 and ginsenoside Rb1 (Rb1) [9].

Our study showed that the combined SalB and Rg1, rather than the combined SalB and Rb1, improved heart contractility in rats with myocardial infarction [10]. SalB holds antioxidant, anti-arteriosclerotic and anti-inflammatory effects and prevents angina pectoris and myocardial ischemia [11,12]. Rg1 possesses anti-fatigue properties and excites the central nervous system [13]. Our recent study elucidated that the SalB-Rg1 combination exerted better cardio-protection than mono-therapy of SalB or Rg1 alone, further indicating the importance to evaluate the safety of the SalB-Rg1 combination [3].

Safety assessment is a key step in drug research and development. To evaluate the preliminary safety of the SalB-Rg1 combination, an acute toxicity and a repeat-dose toxicity study on mice was conducted in the present study.

## 2. Results

### 2.1. Purity of SalB and Rg1

**Figure 1:**
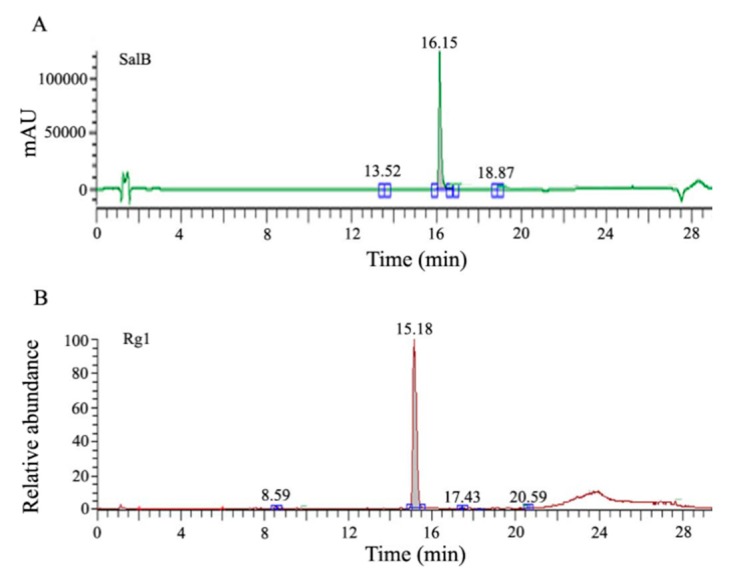
Representative chromatograms of salvianolic acid B (SalB) and ginsenoside Rg1 (Rg1). (**A**) The purity of SalB was 98.46%; and (**B**) the purity of Rg1 was 98.35%.

The purity of SalB and Rg1 was detected by an ultra-high performance liquid chromatography (UHPLC) system. The DAD detection wavelength was set at 280 nm for SalB, and an LTQ Velos Pro mass spectrometer was used as the detector for Rg1. The representative chromatogram for SalB is shown in Figure 1A, and the representative chromatogram for Rg1 is shown in Figure 1B. The purity of SalB was 98.46%, and the purity of Rg1 was 98.35%.

### 2.2. LD_50_ from the Up and Down Procedure

**Figure 2:**
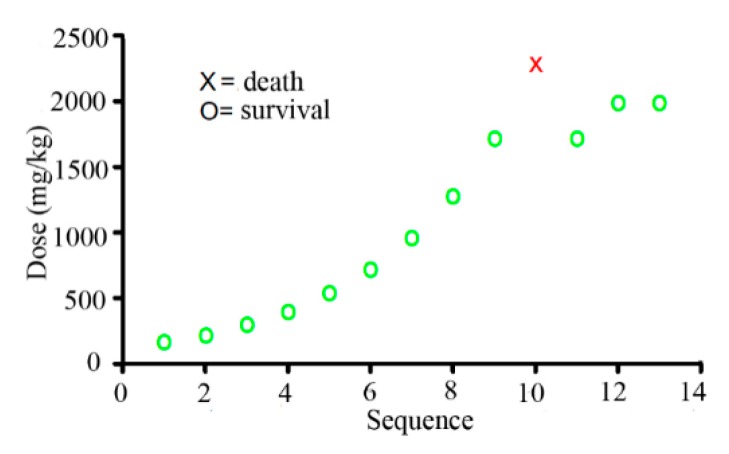
The sequence of the up and down procedure. The surviving animals are marked as green circles and the dead animals as a red cross. The initial dose was 175 mg/kg of the SalB-Rg1 combination.

In the limit test, a female mouse died after being intravenously administrated with a single dose of 2000 mg/kg SalB-Rg1. Then, the main test was performed after. In the main test, one female mouse was randomly selected and administered intravenously with a preliminary dose of 175 mg/kg SalB-Rg1. The next ascending or descending dose was determined depending on the survival from the preceding dose of the animals using a dose progression factor of 1.3. The actual dose progression is shown in Figure 2. With the dose increased to 1290 mg/kg and above, the surviving mice stayed motionless with rapid breathing, which turned back to normal within 2 h. Almost all of the deaths occurred within 20 min after the injection. The estimated LD_50_ was 2560 mg/kg, and the approximated 95% confidence interval was 2000–2300 mg/kg, indicated by the AOT 425 Statistical Program.

### 2.3. LD_50_ from the Bliss Method with a Single Dose

**Figure 3:**
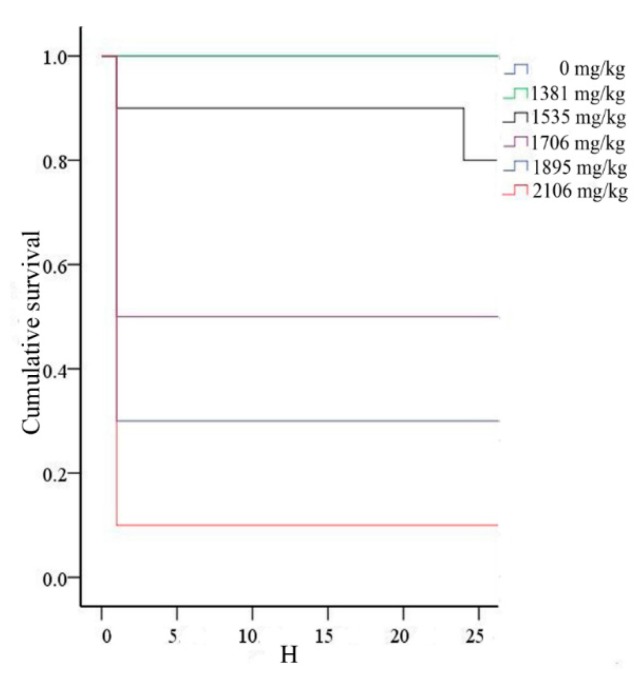
The survival curve for acute toxicity. All of the deaths occurred within 24 h after intravenous administration of SalB-Rg1.

The Bliss method was employed to obtain an accurate value of LD_50_, and the selected dose was based on the results from the up and down procedure. Mice began to die within 24 h after intravenous administration of SalB-Rg1. Additionally, no more mice died during the extended observation days. The percentage of dead mice was 0% (0 mg/kg), 0% (1381 mg/kg), 20% (1535 mg/kg), 50% (1706 mg/kg), 50% (1895 mg/kg) and 90% (2106 mg/kg)), respectively. The cumulative survival curve was drawn based on the observation covering female and male mice within 24 h, and no more mice died thereafter (Figure 3). None of the mice died in the 1381-mg/kg treatment group. The lowest detected dose was 1535 mg/kg for mice that began to die. With the dose increased, the mortality increased (Table 1). There was no significant influence of SalB-Rg1 on body weight during the observation period in the single-dose treatment. According to the Bliss method, the calculated LD_50_ for SalB-Rg1 was 1747 mg/kg with a 95% confidence interval of 1640–1865 mg/kg for intravenous administration.

**Table 1:** Effects of SalB-Rg1 on body weight and survival number with a single dose in acute toxicity.

		Day 0		Day 7		Day 14	
	Dose (mg/kg)	Body Weight (g)	Animal Number	Body Weight (g)	Animal Number	Body Weight (g)	Animal Number
**Males**	0	38.1 ± 2.1	5	42.2 ± 3.3	5	40.6 ± 3.2	5
	1381	37.5 ± 1.9	5	40.6 ± 1.8	5	40.4 ± 1.9	5
	1535	38.5 ± 2.5	5	42.2 ± 3.0	5	42.0 ± 2.9	5
	1706	38.0 ± 2.5	3	40.0 ± 3.3	3	39.5 ± 2.9	3
	1895	37.6 ± 1.2	1	38.7	1	35.7	1
	2106	38.3 ± 2.1	0	–	–	–	–
**Females**	0	32.1 ± 1.2	5	33.8 ± 1.7	5	35.6 ± 1.7	5
	1381	30.5 ± 1.3	5	31.5 ± 1.9	5	33.8 ± 2.1	5
	1535	31.7 ± 2.0	4	33.7 ± 1.9	3	33.4 ± 1.7	3
	1706	30.5 ± 1.6	2	35.2 ± 0.1	2	32.4 ± 0.2	2
	1895	29.9 ± 2.4	2	31.5 ± 2.5	2	32.1 ± 2.1	2
	2106	30.3 ± 1.6	1	30.9	1	31.3	1

Values are expressed as the mean ± SD.

### 2.4. Effects of SalB-Rg1 on the Organ Index with a Single Dose in Acute Toxicity

The effect of SalB-Rg1 on the organ index with a single dose is shown in Table 2. There was no significant variation on the brain, heart, kidney, liver and lung index between the control group and SalB-Rg1-treated groups for both sexes. A significant increase of the spleen index of female mice was observed in the 1535 mg/kg group compared to the control group (4.8 ± 0.6 g/kg *versus* 3.5 ± 0.3 g/kg, *p* < 0.01) and the 1895-mg/kg group compared to the control group (5.8 ± 0.4 g/kg *versus* 3.5 ± 0.3 g/kg, *p* < 0.01). No significant difference on the spleen index was observed in male mice at any detected dose of SalB-Rg1.

**Table 2:** Effects of SalB-Rg1 with a single dose on the organ index in acute toxicity.

Organ Index (g/kg)	Dose Group (mg/kg)					
	0	1381	1535	1706	1895	2106
**Males**						
Brain	8.4 ± 1.5	9.2 ± 0.8	8.9 ± 0.8	8.5 ± 0.3	10.6	–
Heart	5.1 ± 0.2	5.4 ± 0.6	5.6 ± 0.7	4.7 ± 0.7	4.5	–
Kidney	15.4 ± 1.3	15.2 ± 1.3	15.2 ± 0.8	16.7 ± 2.2	17.9	–
Liver	39.9 ± 3.7	45.1 ± 5.4	43.8 ± 1.9	40.1 ± 0.8	44.7	–
Lung	7.1 ± 0.9	7.1 ± 1.0	6.8 ± 1.5	6.6 ± 0.7	7.27	–
Spleen	2.4 ± 0.4	3.7 ± 1.9	2.5 ± 0.6	3.4 ± 1.5	4.5	–
**Females**						
Brain	9.9 ± 1.0	10.8 ± 1.4	11.7 ± 2.6	13.3 ± 1.1	11.1 ± 2.0	11
Heart	4.8 ± 0.4	5.7 ± 0.4	5.8 ± 0.5	5.2 ± 0.4	3.5 ± 0.4	6.6
Kidney	12.6 ± 1.5	13.0 ± 1.4	13.5 ± 2.0	13.3 ± 0.4	13.2 ± 0.2	13.4
Liver	43.7 ± 4.7	46.0 ± 3.9	45.0 ± 3.0	38.5 ± 0.17	50.8 ± 0.8	43.5
Lung	7.22 ± 1.3	6.9 ± 0.7	6.9 ± 1.2	6.9 ± 0.2	7.02 ± 0.5	6.4
Spleen	3.5 ± 0.3	4.1 ± 0.5	4.8 ± 0.6 **	2.6 ± 0.38	5.8 ± 0.4 **	3.7

Values are expressed as the mean ± SD. ** *p* < 0.01 compared to the control group.

**Figure 4:**
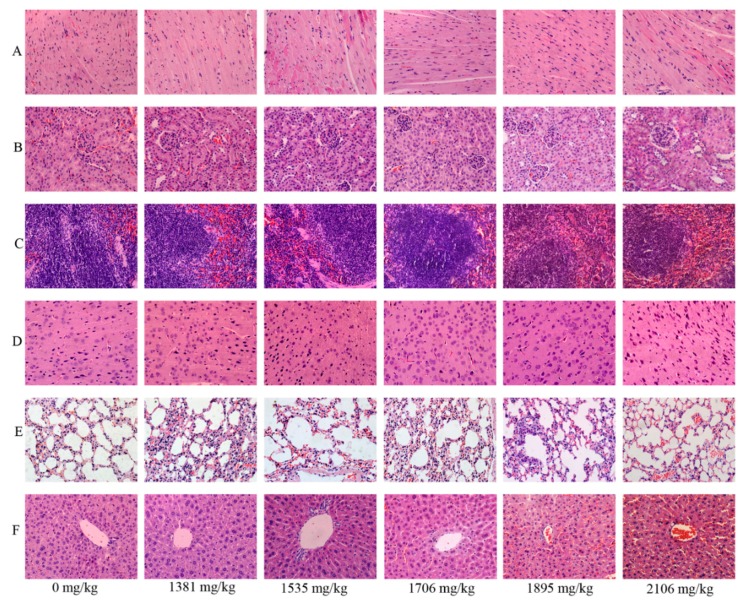
Representative histological pictures of heart (**A**), kidney (**B**), spleen (**C**), brain (**D**), lung (**E**) and liver (**F**) staining with H&E in the acute toxicity study (magnification 400×).

### 2.5. Effects of SalB-Rg1 on the Organ Structure with a Single Dose in Acute Toxicity

The toxicity of SalB-Rg1 with a single dose was further assessed by histological examination. Representative pictures for each organ are manifested at 400× magnification in Figure 4. No abnormality was found in the endocardium, epicardium, myocardium and myocardial interstitial tissue of heart in all treated groups. As for kidney, the structure of renal cortex and medulla was normal, and there was no hyperemia or exudation in the renal corpuscles. Besides, there were no significant morphological alterations detected in spleen, brain and lung in all of the specimens.

### 2.6. Effects of SalB-Rg1 on Biochemical Parameters with a Single Dose in Acute Toxicity

Serum biochemical parameters were determined by commercially-available assay kits according to the instruction of the manufacture (Table 3). No treatment-related serum biochemical variation on creatinine (Cr), total protein (TP) and albumin (Alb) was validated in either sex of mice.

**Table 3:** Effects of SalB-Rg1 with a single dose on the biochemical parameters in acute toxicity.

	Dose Group (mg/kg)					
	0	1381	1535	1706	1895	2106
**Males**						
Cr (μmol/L)	201.2 ± 88.2	159.5 ± 38.8	193.6 ± 12.4	162.3 ± 87.2	–	–
TP (mg/mL)	48.7 ± 2.4	41.2 ± 7.0	38.5 ± 7.5	45.2 ± 10.8	–	–
Alb (mg/mL)	19.2 ± 2.2	19.7 ± 4.6	17.3 ± 3.7	18.6 ± 3.3	–	–
**Females**						
Cr (μmol/L)	125.3 ± 81.3	153.8 ± 58.7	100.6 ± 71.7	323.3 ± 194.6	162.3 ± 33.6	176.5
TP (mg/mL)	44.0 ± 3.7	41.0 ± 2.2	47.5 ± 6.5	47.9 ± 3.0	41.7 ± 6.2	23.2
Alb (mg/mL)	20.2 ± 2.9	18.0 ± 3.8	16.8 ± 1.0	19.7 ± 0.2	17.9 ± 3.2	47.8

Values are expressed as the mean ± SD. Cr: creatinine; TP; total protein; Alb: albumin.

### 2.7. Effects of SalB-Rg1 on Body Weight and Survival with a Seven-Day Repeated Dose for Toxicity Evaluation

In our previous study, an effective dose for cardio-protection was used (15 mg/kg). In the present study, we evaluated the toxicity for a seven-day repeated dose of 15, 30 and 60 mg/kg. During the observation period of seven days, a survival rate of 100% was obtained. There was also no significant influence of SalB-Rg1 on body weight (Table 4).

**Table 4:** Effects of SalB-Rg1 on body weight and survival number with seven-day repeated dose toxicity.

		Day 0		Day 7		Day 14	
	Dose (mg/kg)	Body Weight (g)	Animal Number	Body Weight (g)	Animal Number	Body Weight (g)	Animal Number
**Males**	0	23.5 ± 1.1	5	27.4 ± 1.5	5	30.8 ± 2.2	5
	15	23.5 ± 1.1	5	27.4 ± 1.5	5	30.8 ± 2.2	5
	30	22.8 ± 0.8	5	26.4 ± 1.1	5	30.0 ± 1.5	5
	60	23.1 ± 0.9	5	27.0 ± 1.3	5	30.0 ± 1.3	5
**Females**	0	22.2 ± 1.2	5	24.7 ± 1.3	5	26.6 ± 1.1	5
	15	22.9 ± 0.7	5	26.9 ± 1.0	5	26.8 ± 1.2	5
	30	23.0 ± 0.7	5	26.6 ± 2.2	5	28.5 ± 1.2	5
	60	22.2 ± 1.5	5	25.7 ± 1.9	5	26.7 ± 1.7	5

Values are expressed as the mean ± SD.

### 2.8. Effects of SalB-Rg1 on the Organ Index with a Seven-Day Repeated Dose for Toxicity Evaluation

The effect of SalB-Rg1 on the organ index with a seven-day repeated dose is shown in Table 5. No significant difference on the organ index, including brain, heart, kidney, spleen and lung, comparing the control group and SalB-Rg1 group with the indicated dose, was observed in either the male or female mice.

**Table 5:** Effects of SalB-Rg1 on the organ index with a seven-day repeated dose.

Organ Index (g/kg)	Dose Group (mg/kg)			
	0	15	30	60
**Males**				
Brain	11.5 ± 1.2	11.6 ± 1.1	11.8 ± 1.3	11.7 ± 1.1
Heart	5.7 ± 0.5	5.2 ± 0.3	5.8 ± 0.5	5.5 ± 0.5
Kidney	14.0 ± 0.7	13.6 ± 0.6	13.0 ± 1.3	13.6 ± 0.9
Lung	7.3 ± 0.4	6.6 ± 0.4	7.0 ± 0.5	6.8 ± 0.6
Spleen	8.3 ± 3.4	6.4 ± 0.4	6.8 ± 0.7	6.1 ± 0.4
**Females**				
Brain	13.4 ± 1.7	12.6 ± 1.0	12.1 ± 1.3	13.6 ± 0.5
Heart	5.1 ± 0.5	4.9 ± 0.2	4.9 ± 0.5	4.8 ± 0.5
Kidney	13.3 ± 0.6	13.5 ± 1.1	13.4 ± 0.9	12.4 ± 0.9
Lung	8.2 ± 1.8	7.5 ± 0.7	6.8 ± 1.1	7.4 ± 1.2
Spleen	7.3 ± 0.9	6.5 ± 1.0	5.9 ± 1.2	6.3 ± 1.2

Values are expressed as the mean ± SD.

### 2.9. Effects of SalB-Rg1 on the Organ Structure with a Seven-Day Repeated Dose for Toxicity Evaluation

**Figure 5:**
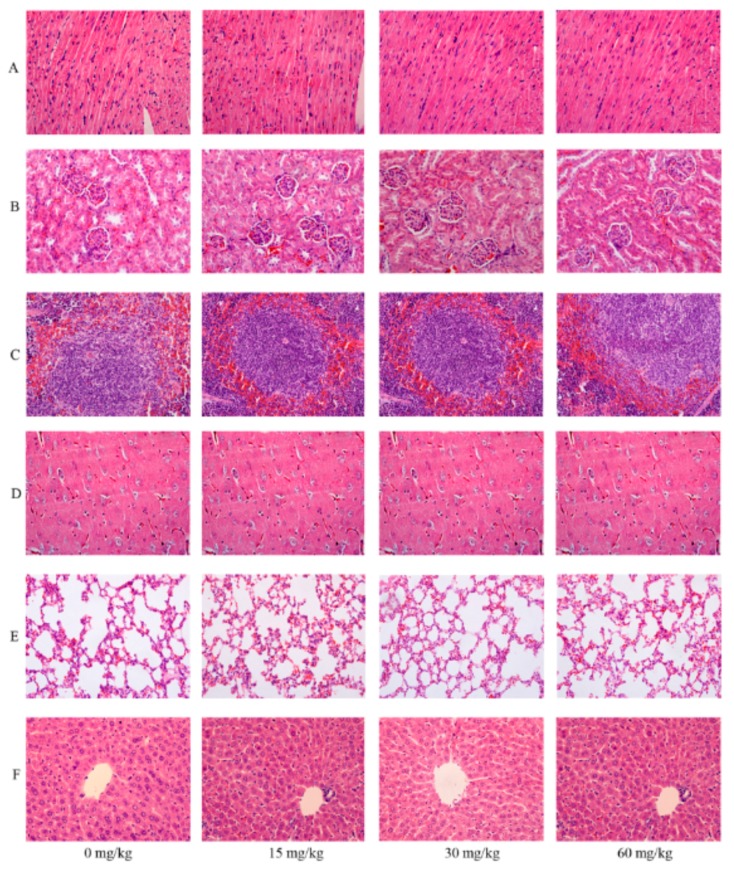
Representative histological pictures of heart (**A**), kidney (**B**), spleen (**C**), brain (**D**), lung (**E**) and liver (**F**) staining with H&E in the seven-day repeated toxicity study (magnification 400×).

Pathological examination of SalB-Rg1 with a seven-day repeated dose study was also assessed using H&E stain. Representative pictures for various tissue structures are manifested in Figure 5 at 400× magnification. The absence of abnormality was found on heart, liver, spleen, lung, kidney and brain with SalB-Rg1 treatment at different doses.

### 2.10. Effects of SalB-Rg1 on Biochemical Parameters of the Seven-Day Repeated Dose for Toxicity Evaluation

The serum concentration of biochemical parameter was also conducted to assess toxicity of SalB-Rg1 with a seven-day repeated dose (Table 6). No treatment-related serum biochemical variation on TP and Alb was found in either sex of mice. For the female mice, 60 mg/kg of SalB-Rg1 downregulated the value of Cr compared to the control group (44.5 ± 20.6 μmol/L *versus* 81.5 ± 14.7 μmol/L), while SalB-Rg1 did not reveal any influence on the value of Cr in male mice.

**Table 6:** Effects of SalB-Rg1 on biochemical parameters with a seven-day repeat dose.

	Dose Group (mg/kg)			
	0	15	30	60
**Males**				
Cr (μmol/L)	76.3 ± 49.8	38.7 ± 22.0	67.1 ± 11.3	58.9 ± 12.3
TP (mg/mL)	52.3 ± 5.4	56.1 ± 5.4	52.4 ± 4.9	52.7 ± 5.6
Alb (mg/mL)	28.0 ± 3.2	29.6 ± 1.7	28.5 ± 4.9	28.6 ± 3.1
**Females**				
Cr (μmol/L)	81.5 ± 14.7	81.5 ± 10.6	70.2 ± 17.4	44.5 ± 20.6 *
TP (mg/mL)	54.3 ± 3.9	56.4 ± 4.9	54.1 ± 6.9	53.1 ± 4.6
Alb (mg/mL)	28.7 ± 3.3	28.7 ± 1.8	29.2 ± 3.5	33.3 ± 3.9

Values are the mean ± SD. Cr: creatinine, TP: total protein, Alb: albumin. Compared to the control group: * *p* < 0.05.

## 3. Discussion

To evaluate the safety of the SalB-Rg1 combination, we examined acute toxicity and seven-day repeated dose toxicity in mice of both sexes. The mortality and alteration in gross observation, body weight, organ index, serum biochemistry and histopathology were monitored. The LD_50_ of the SalB-Rg1 combination was 1747 mg/kg for intravenous administration. No obvious toxicity was observed for both studies. 

The therapeutic window is the range of drug dosages that can treat disease effectively while staying within the safety range. Medication with a narrow therapeutic window must be administered with care to avoid irreversible damage [14,15]. Even though we did not perform a rigorous study to evaluate the therapeutic window for SalB-Rg1, the obtained intravenous LD_50_ of SalB-Rg1 was 1747 mg/kg, which was 100-times higher than the effective dose (15 mg/kg), indicating the wide safety range of SalB-Rg1.

Danshen, the dried root of *Salvia miltiorrhiza*, has been widely used in China, to a lesser extent in Japan, the United States and European countries, for the treatment of cardiovascular and cerebrovascular diseases [16]. Sanqi is a Chinese herbal medicine prepared from the roots of the herb *Panax notoginseng* [2]. The Danshen dripping pill is a popular Chinese prescription in treating coronary heart disease and angina pectoris [17]. *Salvia miltiorrhiza* and *Panax notoginseng* are the main herb composition in the Danshen dripping pill. The oral LD_50_ tested on the mice for a water-soluble extract of the Danshen dripping pill was 25.807 g/kg, which was equivalent to 3934-times the human oral dose (6.56 mg/kg) [18]. These results are consistent with our present findings, indicating the safety of combined *Salvia miltiorrhiza* and *Panax notoginseng* at both the extract level and the compound level.

In our acute toxicity study, mice with instant death appeared motionless, indicating the possible influence on the central nervous system. The other surviving mice returned to normal within 1 h. It seemed that the adverse stimulation of the central nervous system was reversible. Another phenomenon was observed that the mice began to struggle when the dose increased to 1535 mg/kg, while no struggling was observed on mice for the seven-day repeated toxicity, suggesting that this adverse effect was dose dependent. Commonly, abnormal alterations to body weight in toxicity studies have been regarded as critical indicators of adverse effects [19]. No significant alteration to body weight was found, both in the acute and repeated dose study at all doses, implying that SalB-Rg1 was well tolerated by the mice.

On the other hand, the spleen index increased in the treatment group with the doses of 1535 and 1895 mg/kg for female mice in the acute toxicity study, while a similar increase was not found in the study of seven-day repeated dose toxicity, suggesting that the influence of SalB-Rg1 on spleen was dose related. Nevertheless, the specific mechanism for the influence of SalB-Rg1 on the spleen index should be further elucidated.

In conclusion, the LD_50_ in mice was 1747 mg/kg, which was one hundred-times higher than the effective dose (15 mg/kg). Both studies of acute toxicity and seven-day repeated dose toxicity indicated the safety of the SalB-Rg1 combination. Therefore, our results suggest that SalB-Rg1 was a safe combination for further development.

## 4. Experimental Section

### 4.1. Reagents

SalB and Rg1 were purchased from Shanghai Yousi Bio-Tech Co., Ltd (Shanghai, China). The structure of SalB or Rg1 was elucidated by the ^1^H and ^13^C NMR spectrum using a Bruker AM-400 spectrometer (data not shown). All of the other reagents were commercially purchased from DingGuo (Shanghai, China), unless specified otherwise. The protective effects of the SalB-Rg1 combination with various ratios were evaluated by several major hemodynamic parameters for left ventricle function, and the optimized ratio for SalB to Rg1 was 2:5 based on our previous data [3] (Figure S1). Consequently in the present studies, the SalB-Rg1 combination at a ratio of 2:5 was employed to evaluate its toxicity.

### 4.2. Animals

This study was approved by the Animal Care and Use Committee at Shanghai Institute of Materia Medica (IACUC number: SIMM-2013-08-GDA-19), and all experiments were performed according to the Guide for the Care and Use of Laboratory Animals published by the National Institutes of Health. Healthy female and male Kunming mice were obtained from Shanghai Center of Experimental Animals, Chinese Academy of Sciences, and kept in a temperature-controlled room (22 ± 2 °C) with a 12-h light and dark cycle. Water and diet were available *ad libitum*. The mice were acclimatized for two weeks prior to the test.

### 4.3. Purity Assay for SalB and Rg1

The purity of SalB and Rg1 was detected by an ultra-high performance liquid chromatography (UHPLC) system. Briefly, the compound solution was filtered through a 0.22-μm membrane and injected into the UltiMate 3000 Binary RSLC system (Thermo Fisher Scientific Inc., Waltham, MA, USA). The column configuration consisted of a Zorbax Eclipse Plus C18 column (1.8 μm, 100 mm × 2.1 mm) and a Zorbax Eclipse Plus C18 Guard column (1.8 μm, 5 mm × 2.1 mm). The sample injection volume was 2 μL. The DAD detection wavelength was set at 280 nm for SalB, and an LTQ Velos Pro mass spectrometer was used as the detector for Rg1; the flow rate was 0.21 mL/min, and the column temperature was maintained at 25 °C. The mobile phase consisted of (A) acetonitrile and (B) 0.1% aqueous formic acid (V/V), using a gradient elution of 10%–20% A at 0–6 min, 20%–25% A at 6–14 min, 25%–30% A at 14–18 min, 30%–90% A at 18–22 min, then holding at 90% A for 3 min.

### 4.4. Up and Down Procedure

Acute single-dose toxicity was assessed on the basis of mortality. Firstly, the limit test and the main test were performed following the up and down procedure of the OECD Guidelines for Testing of Chemicals No. 425 (OECD, 2006) on female Kunming mice. The state of survival and death was recorded after dosing and then kept for a further 14 days with a once daily observation for surviving mice. Body weight was recorded before dosing and at weekly intervals thereafter. At the end of the experiment, all surviving animals were sacrificed, and the visceral organs were examined.

For the limit test, a single limit dose of 2000 mg/kg of SalB-Rg1 was dissolved in normal saline and intravenously administered. The animal was observed, and gross necropsy was performed on the dead animal after a 2000-mg/kg SalB-Rg1 administration. Because the treated animals died in the limit test, the main test was performed as follows. In the main test, one female mouse was randomly selected and administered intravenously with a preliminary dose of 175 mg/kg of SalB-Rg1. The general behavior of the animals was monitored for 24 h after treatment and thereafter daily up to 14 days. The next ascending or descending doses were determined depending on the survival from the preceding dose of the animals. The acute toxicity test for calculating the median lethal dose (LD_50_) and the 95% confidence interval were calculated using the AOT 425 Statistical Program.

### 4.5. Bliss Method

After the up and down procedure, sixty Kunming mice were randomly divided into six groups (*n* = 10, male and female in half). Control mice received normal saline; the other five groups received graded doses (1381, 1535, 1706, 1895 and 2106 mg/kg) of SalB-Rg1. A single dose of SalB-Rg1 was administrated intravenously at a volume of 10 mL/kg body weight. The animals were observed for gross signs of toxicity (respiratory system, neurologic system, motor function and gastrointestinal system) and mortality for 24 h and then daily for a further 14 days. The LD_50_ was calculated following the SPSS analysis system based on the mortality throughout the study.

### 4.6. Seven-Day Repeat Dose

To evaluate the toxicity of SalB-Rg1 at the effective dose for therapy, mice were divided randomly into 4 groups (*n* = 10, male and female in half). Control mice received normal saline; the other three groups received graded doses (15, 30, 60 mg/kg) of SalB-Rg1 intravenously once a day for 1 week; then, mice were scarified, and the structure of the main organs (heart, liver, spleen, brain, kidney and lung) was evaluated by histopathological examination. Serum biochemical parameters were evaluated by a commercial kit according to the description of the manufacture.

### 4.7. Collection of Blood and Organ Samples

After toxic observation, the mice were anaesthetized with choral hydrate (350 mg/kg). Blood samples were collected, and serum was separated by centrifugation at 2000× *g* for 15 min at 4 °C, then stored at −80 °C until the time of the assay. Heart, liver, spleen, brain, kidney and lung were dissected out, weighed and fixed for further examination. The organ index was calculated using the equation that organ weight (g) was divided by body weight (kg).

### 4.8. Histopathological Detection

The main organ samples (heart, liver, spleen, brain, kidney and lung) were fixed by 4% neutral-buffered paraformaldehyde for 24 h, and the specimens were paraffin-embedded, cut at 3 μm and stained with hematoxylin and eosin. Photomicrographs were taken using an Olympus BX51 microscope plus an Olympus DP71 CCD camera (Olympus Corporation, Tokyo, Japan).

### 4.9. Measurement of Biochemical Parameters on Serum

Serum concentrations of creatinine (Cr), total protein (TP) and albumin (Alb) were determined by commercially-available assay kits (Nanjing Jiancheng Bioengineering Institute, Nanjing, China). Optical density was recorded for Cr at 510 nm, TP at 540 nm and Alb at 510 nm using a microplate reader (TecanGENios, Männedorf, Austria). The concentration of each parameter was calculated according to the manufacturer’s instruction.

### 4.10. Statistical Analysis

Statistical analysis was performed by SPSS software. The LD_50_ from the Bliss method was calculated according to the weighted probit regression method. All quantitative values were given as the mean ± SD and analyzed using one-way analysis of variance (ANOVA) following Dunnett’s *t*-test. *p* < 0.05 was considered to be statistically significant.

## Supplementary Materials

Supplementary materials can be found at http://www.mdpi.com/1422-0067/16/12/26176/s1.
