# Supplementary material for: The Safety Evaluation of Salvianolic Acid B and Ginsenoside Rg1 Combination on Mice
Source: Int J Mol Sci. 2015 Dec 9;16(12):29345–56. doi: 10.3390/ijms161226176 (PMC4691119; doi:10.3390/ijms161226176)
Supplement: Supplementary file 1 [file ijms-16-26176-s001.pdf]

Supplementary Materials: The Safety Evaluation of Salvianolic Acid B and Ginsenoside Rg1 Combination on Mice

Qun Zhao, Min Yang, Yanping Deng, Haitao Yu, Linlin Wang, FukangTeng, Kenka Cho, Hongmei Ma, Peng Wu, Xue Li, Wanying Wu, Xuan Liu, Feng Xu, Baohong Jiang and De-An Guo

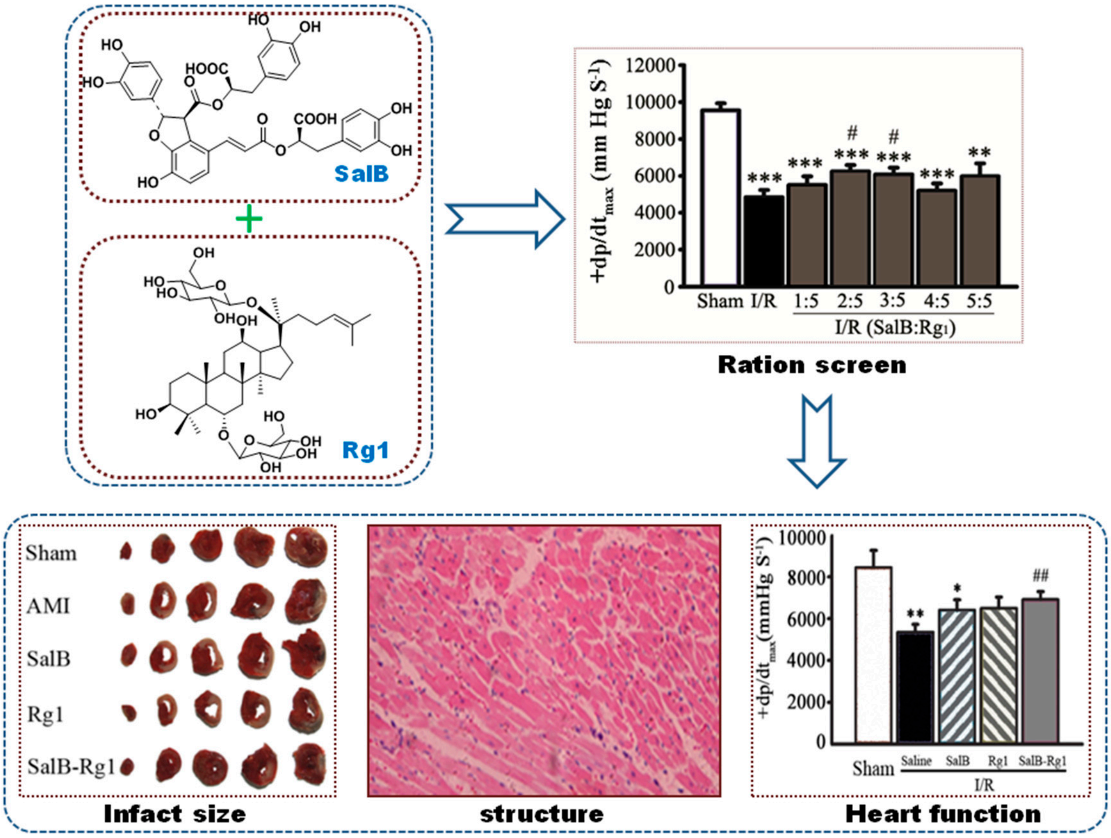

Figure S1. The ratio for SalB to Rg1 was set as 2:5 based on ventricular function. SalB-Rg1 (2:5) improved cardiac function and protected cardiac structure in rats with ischemia/reperfusion injury [3]. Reprinted with permission from [3]. * $p < 0.05$, ** $p < 0.01$ and *** $p < 0.001$ compared to the sham group; # $p < 0.05$, ## $p < 0.01$ compared to the I/R group (Ration screen and Heart function).
